# Effects of Antimicrobial Underwear on Vaginal Symptoms and Vaginal Microbiota: A Prospective Study

**DOI:** 10.3390/jcm15041395

**Published:** 2026-02-10

**Authors:** Yunus Öztoprak, Metehan Öztoprak, Emre Destegül, Sefa Arlıer, Atahan Töre Toklu, Hasan Can Toyganözü, Cevdet Adıgüzel

**Affiliations:** 1 Department of Obstetrics and Gynecology, Ceylanpınar State Hospital, Şanlıurfa 63570, Turkey; 2 Medicaltex, Kayseri 38030, Turkey; metehanoztoprak@gmail.com; 3 Department of Obstetrics and Gynecology, Private Adana Middle East Hospital, Adana 01140, Turkey; destegulemre@hotmail.com; 4 Department of Obstetrics and Gynecology, Adana City Training and Research Hospital, Adana 01120, Turkey; sefaarlier@gmail.com (S.A.); atahantore@gmail.com (A.T.T.); 5 Department of Obstetrics and Gynecology, Private Medline Adana Hospital, Adana 01170, Turkey; hasanbaskent@hotmail.com; 6 Department of Obstetrics and Gynecology, Medical Park Adana Hospital, Adana 01160, Turkey; cevdetadiguzel@yahoo.com

**Keywords:** vaginitis, antimicrobial underwear, textile technology, microbiological clearance

## Abstract

**Objective:** To evaluate the clinical and microbiological effects of antimicrobial underwear as an adjunct to standard treatment in women with acute vaginitis. **Methods:** Sixty reproductive-age women with acute vaginitis received a 7-day intravaginal regimen of metronidazole and miconazole. Participants were assigned either to a group wearing antimicrobial underwear or to a control group wearing non-antimicrobial underwear. Vaginal symptoms and culture results were assessed before and after treatment. **Results:** The antimicrobial-underwear group showed significant improvement in vaginal symptoms, including discharge (96.7%→6.9% vs. 72.5%→27.5%; *p* < 0.001), pruritus (37.5% vs. 68.4%; *p* = 0.044), odor (19.6% vs. 53.8%; *p* = 0.016), and irritation (36.4% vs. 75%; *p* = 0.013). Dyspareunia was similar between groups. While no microbiological change was observed in controls (*p* = 0.950), negative cultures increased from 40% to 80% in the antimicrobial-underwear group (*p* = 0.018), with marked reductions in *Candida* spp., *Gardnerella vaginalis*, *E. coli*, and *Klebsiella* spp. **Conclusions:** Antimicrobial underwear, when used in conjunction with standard treatment, can enhance symptom relief and maintain genital hygiene. By improving the vulvovaginal microenvironment, antimicrobial textiles can reduce moisture and the persistence of pathogens.

## 1. Introduction

Vaginal health is shaped by a complex interplay of hormonal, immunological, and microbial factors, and disturbances in this balance may lead to symptoms such as discharge, pruritus, odor, irritation, and dyspareunia, all of which substantially impair quality of life in reproductive-age women [1]. Disruption of this microbial equilibrium—whether through behavioral, environmental, or iatrogenic factors—has been associated with increased susceptibility to bacterial vaginosis, vulvovaginal candidiasis, and mixed aerobic–anaerobic infections [2,3].

Several modifiable lifestyle factors have been implicated in shaping the vaginal microenvironment, including personal hygiene practices, sexual behavior, menstrual hygiene products, and clothing habits [4]. In particular, underwear type and textile composition have emerged as potential determinants of moisture retention, friction, local temperature, and microbial colonization in the vulvovaginal region [5]. Synthetic fabrics may create a warm, humid microenvironment that promotes dysbiosis, thereby facilitating colonization by *Candida* species and anaerobic bacteria [6].

In response to these concerns, antimicrobial textiles incorporating bioactive fibers—such as silver ions, copper oxide, or chitosan—have been developed with the aim of reducing bacterial and fungal growth at the skin–fabric interface [7,8]. Studies in dermatology and material science demonstrate that such fabrics may reduce microbial burden, humidity, and irritation, thus potentially contributing to improved genital comfort [9,10]. However, despite widespread commercial promotion of antimicrobial underwear, empirical evidence evaluating their effects specifically on vaginal symptoms and vaginal microbiota remains limited. Only a small number of recent clinical studies have explored the microbiological and symptomatic outcomes associated with antimicrobial fabric use, leaving a significant gap in the literature regarding their clinical relevance [8,9,10].

Therefore, the present study aimed to assess the effects of antimicrobial underwear on vaginal symptoms and vaginal microbiota in reproductive-age women. By comparing clinical symptoms and culture-based microbiological outcomes before and after antimicrobial underwear use, and contrasting them with a non-underwear control group, this study provides new evidence regarding the potential role of antimicrobial textiles in gynecological practice.

## 2. Materials and Methods

This prospective study included 60 women of reproductive age diagnosed with acute vaginitis who presented to the gynecology outpatient clinic between June 2025 and September 2025. Participants were randomly assigned to two groups with computer: one group wearing antimicrobial underwear (*n* = 30) and one control group wearing non-antimicrobial underwear (*n* = 30). Patients with acute vaginitis who were sexually active, nonpregnant, and had no systemic infection were included in the study. Exclusion criteria included recent antibiotic or antifungal use, vulvar dermatologic disease, immunosuppression, intravenous device use, and active other infectious disease. Ethics approval was obtained from the Institutional Ethics Committee (Approval No: 567), and all participants provided written informed consent.

All patients participating in the study received a 7-day course of vaginal suppositories containing 750 mg metronidazole and 200 mg miconazole for acute vaginitis. As an adjunct to this treatment, women in the intervention group were given antibacterial underwear containing antimicrobial textile fibers designed to inhibit bacterial and fungal growth. During the study, women in the study group were instructed to wear antimicrobial underwear daily, while women in the control group were instructed to wear their own non-antimicrobial cotton underwear. All participants continued their usual hygiene practices.

Patients’ age, body mass index (BMI), hemoglobin (Hb), C-reactive protein (CRP), white blood cell (WBC) count, and procalcitonin (PCT) levels were recorded. Patients were asked about their complaints of vaginal discharge, itching, odor, irritation, and dyspareunia before and after treatment, and these were recorded and evaluated. Vaginal swab samples were obtained from the posterior fornix of the vagina using sterile cotton swabs under appropriate aseptic conditions before and after treatment. The samples were immediately transported to the mycology laboratory and inoculated onto Sabouraud dextrose agar (SDA) media. The culture plates were incubated under aerobic conditions at 35–37 °C for 24–48 h. At the end of the incubation period, the plates were evaluated for fungal growth, colony morphology, and growth characteristics. Pure cultures were obtained from the growing yeast colonies and reserved for further identification procedures. Species-level identification of all isolates was performed using the MALDI-TOF MS method (Bruker Daltonics, Bremen, Germany).

Antimicrobial underwear textiles are produced as follows: The ammonium quaternary compounds are applied to the textile at a 0.1% flotte ratio using a proprietary process developed by our company. This process ensures strong binding of the active components to the fabric surface.

### Statistical Analysis

All statistical analyses were performed using IBM SPSS Statistics, version 26 (IBM Corp., Armonk, NY, USA). We used the Shapiro–Wilk test to determine whether continuous data were normally distributed. While the mean ± standard deviation was used for normally distributed continuous variables, the median [25–75%] was used for other variables. Categorical variables are presented as numbers and percentages. Categorical data were compared using Chi-square or Fisher’s exact test. For the comparison of two independent groups, the Mann–Whitney U test was used if the distribution was not normal, and the independent sample *t* test was used if the distribution was normal. We accepted *p* < 0.05 as statistically significant.

## 3. Results

No statistically significant differences were observed between the two groups in terms of demographic characteristics and laboratory results such as age, BMI, Hb concentration, white blood cell count, CRP levels and PCT values (*p* > 0.05), indicating that both cohorts were clinically similar at study entry (*p* > 0.05) (Table 1).

At baseline, the frequency of vaginal symptoms was generally similar between the groups. The prevalence of vaginal discharge, itching, irritation, and dyspareunia did not differ significantly between groups (*p* > 0.05). However, the prevalence of vaginal odor was 73.3% in the study group compared to 40% in the control group, a statistically significant difference (*p* = 0.018) (Table 2).

Significant improvements in vaginal symptoms were observed, particularly in the group of antimicrobial underwear. The proportion of women reporting vaginal discharge decreased significantly, from 96.7% at baseline to only 6.9% after using the antimicrobial underwear. In contrast, the control group also showed improvement (from 72.5% to 27.5%), but the magnitude of change was significantly smaller, resulting in a highly significant difference between the groups (*p* < 0.001). Similarly, the prevalence of vaginal pruritus was significantly lower among women using antimicrobial underwear compared with controls (37.5% vs. 68.4%, *p* = 0.044). A comparable pattern was observed for vaginal odor (19.6% vs. 53.8%, *p* = 0.016) and vaginal irritation (36.4% vs. 75%, *p* = 0.013). These improvements collectively indicate that antimicrobial underwear use led to pronounced symptomatic relief across multiple domains. Dyspareunia was the only symptom that did not differ significantly between the groups (*p* = 0.15) (Table 3).

Among women who used non-antimicrobial underwear, no significant differences were detected between baseline and follow-up vaginal cultures (*p* = 0.950). The proportion of negative cultures remained relatively stable (53.3% at baseline vs. 56.6% at follow-up). Similarly, the distribution of isolated organisms—primarily *Candida albicans*, *C. glabrata*, *E. coli*, and *G. vaginalis*—did not exhibit meaningful variation over time. Mixed microbial growth patterns also persisted in similar proportions in both sampling periods (Table 4).

In contrast, the use of antibacterial underwear resulted in a marked shift in microbiological cultures (*p* = 0.018). The proportion of negative cultures increased from 40% at baseline to 80% after the intervention, indicating a substantial reduction in detect xable pathogenic or opportunistic microorganisms. Organisms commonly identified at baseline—including *C. albicans* (33.3%), *C. glabrata*, *C. krusei*, *E. coli*, *Klebsiella* species, and mixed growth combinations—were either completely absent or significantly reduced in the post-intervention cultures (Table 5).

## 4. Discussion

The present study demonstrates that the use of antimicrobial underwear as an adjunct to standard vaginitis treatment leads to significant improvements in both clinical symptoms and microbiological outcomes in reproductive-age women. After completing a standard 7-day course of metronidazole and miconazole, participants using antimicrobial underwear showed markedly greater reductions in vaginal discharge, pruritus, odor, and irritation compared with the control group, which wore non-antimicrobial underwear. These findings indicate that antimicrobial textile technology may play a meaningful role in restoring the vulvovaginal microenvironment after acute vaginitis treatment. Leveraging non-migrating technology, the chemical is engineered to remain only on the surface. Thanks to the new-generation application method, it provides high efficacy while maintaining an optimal cost structure. Through this specialized process, the chemical forms covalent bonds with the fabric surface, significantly increasing durability and preventing any release of the active compounds. Since no migration or leaching occurs, the product does not disrupt the genital microbiota, ensuring safe and comfortable use.

Vaginal health is increasingly recognized as a delicate balance between microbiota, mucosal immunity, epithelial integrity, and environmental exposures [1]. Disruption of this balance can lead to recurrent or persistent symptoms. Although antimicrobial drug therapy targets pathogens directly, reinfection or recurrence is often driven by environmental or behavioral factors that alter vaginal ecology [2,3]. A study by Côra et al. found that women who wear underwear for most of the day have a higher risk of sexually transmitted infections [5]. The type of textile used in underwear can also cause vaginal infections. One study reported that nylon absorbs less sweat than cotton underwear, moisturizes the groin area, and increases the risk of reproductive tract infections; therefore, women should be careful when choosing the type of fabric for daily use [11]. Furthermore, a systematic review of the literature indicates that underwear is a critical factor in preventing vulvovaginitis. Bacteria and yeasts thrive in moist or wet environments. Therefore, underwear use may contribute to vulvovaginitis [12]. In this context, underwear fabric composition is emerging as a modifiable determinant of vaginal microbial stability. Recent evidence shows that moisture retention, friction, and heat accumulation caused by tight or synthetic fabrics can promote dysbiosis and impair mucosal defense [4,6,10]. Synthetic fabrics are known to create a warm, humid environment favorable to the proliferation of *Candida* species and anaerobic bacteria [6].

In our study, antimicrobial underwear users demonstrated a substantial shift in microbiological patterns: negative cultures doubled from 40% to 80%. Organisms commonly associated with recurrent vaginitis—*C. albicans*, *C. glabrata*, *G. vaginalis*, *E. coli*, *Klebsiella* spp.—were either absent or dramatically reduced in the post-treatment period. This result aligns with the growing body of research showing that the incorporation of quaternary ammonium compounds, nanoparticles, or metallic oxides into textiles inhibits bacterial replication, suppresses fungal growth, and prevents biofilm formation at the fabric–skin interface [7,8,9]. Biofilm-associated communities are less susceptible to metronidazole and azole antifungals, contributing to persistent or recurrent symptoms after treatment [13]. Antimicrobial textiles have demonstrated efficacy in reducing both bacterial burden and humidity in dermatologic applications, which likely contributes to improved genital comfort and reduced irritation [9,10].

Dermatological research indicates that textiles in prolonged skin contact can significantly impact microbial colonization, hydration, pH, and inflammation [14]. Cotton and breathable fabrics reduce moisture retention and promote healthier microbial profiles compared to synthetic materials, which increase transepidermal water loss and irritation [15]. The covalent bonding and non-migrating technology described in the underwear manufacturing process further strengthens the plausibility of this mechanism. Because the active compounds do not leach, they maintain local antimicrobial effects without disrupting the endogenous vaginal microbiota or causing systemic absorption. This is consistent with modern antimicrobial textile research emphasizing durable, non-toxic, surface-bound biocides that act locally [16,17]. Textile bioengineering studies also indicate that antimicrobial fabrics reduce local moisture, suppress microbial transfer between skin and fabric, and reduce irritation in occlusive areas such as the groin [9]. These mechanisms likely contributed to the significant improvements in itch and irritation observed in our cohort. Environmental stabilization may also promote the reestablishment of Lactobacillus-dominated communities by reducing competitive pressure from pathogenic and opportunistic organisms [18].

Collectively, the findings of our study align with contemporary models of vaginal ecology that emphasize the role of non-pharmacologic factors in shaping the healing trajectory. Antimicrobial underwear appears to enhance the effectiveness of standard vaginitis treatment by improving local environmental parameters—reducing moisture, friction, and microbial persistence—thus lowering the likelihood of reinoculation and facilitating mucosal recovery. These insights underscore the potential integration of biomedical textiles into gynecologic practice as adjunctive tools to reduce recurrence and improve symptom outcomes. Future studies incorporating molecular microbiome sequencing, humidity and temperature microenvironment mapping, and randomized controlled comparisons of antimicrobial textile technologies will be essential to delineate their optimal clinical applications.

This study has several limitations that should be acknowledged. First, the sample size was relatively small and the study was conducted at a single center, which may limit the generalizability of the findings. Second, the follow-up period was limited to the short-term post-treatment phase, and long-term outcomes such as recurrence rates of vaginitis were not evaluated. Additionally, adherence to underwear use and hygiene practices was based on patient self-report, which may introduce reporting bias. Finally, because all participants received standard antimicrobial therapy, the isolated effect of antimicrobial underwear alone could not be assessed. Future multicenter studies with larger cohorts, longer follow-up periods, and molecular microbiome analyses are warranted to further clarify the clinical and microbiological impact of antimicrobial textiles in vaginal health.

## Figures and Tables

**Table 1:** Comparison of patient characteristics and clinical features.

Variables	Antimicrobial Underwear Use (*n* = 30)	Non-Antimicrobial Underwear Use (*n* = 30)	*p* Value
Age (years)	35.77 ± 9.49	34.63 ± 8.22	0.622
BMI	23.96 ± 2.92	24.95 ± 2.81	0.185
Hb (g/dL)	12.27 ± 1.51	12.52 ± 1.18	0.479
WBC count	7.98 ± 2.40	8.27 ± 2.05	0.626
CRP (mg/dL)	2 (1.7–2.85)	2.2 (1.95–5.3)	0.243
Procalcitonin (ng/mL)	0.020 (0.010–0.022)	0.020 (0.010–0.020)	0.770

Abbreviations: BMI: body mass index; g/dL: grams per decilitre; mg/dL: milligrams per decilitre; ng/mL: nanograms per millilitre; Hb: haemoglobin; WBC: white blood cell; CRP: c-reactive protein.

**Table 2:** Comparison of vaginal symptoms before underwear uses.

Variables	Antimicrobial Underwear Use (*n* = 30)	Non-Antimicrobial Underwear Use (*n* = 30)	*p* Value
Vaginal discharge *n* (%)			
(+)	29 (96.7%)	29 (96.7%)	0.734
(−)	1 (3.3%)	1 (3.3%)	
Vaginal pruritus *n* (%)			
(+)	24 (80%)	18 (60%)	0.152
(−)	6 (20%)	12 (40%)	
Vaginal odor *n* (%)			
(+)	22 (73.3%)	12 (40%)	0.018
(−)	8 (26.7%)	18 (60%)	
Vaginal irritation *n* (%)			
(+)	22 (73.3%)	20 (66.7%)	0.779
(−)	8 (26.7%)	10 (33.3%)	
Dyspareunia *n* (%)			
(+)	27 (90%)	28 (93.3%)	0.634
(−)	3(10%)	2 (6.7%)	

**Table 3:** Comparison of vaginal symptoms after underwear uses.

Variables	Antimicrobial Underwear Use (*n* = 30)	Non-Antimicrobial Underwear Use (*n* = 30)	*p* Value
Vaginal discharge *n* (%)			<0.001
(+)	2 (6.9%)	21 (72.5%)	
(−)	27 (93.1%)	8 (27.5%)	
Vaginal pruritus *n* (%)			0.044
(+)	9 (37.5%)	13 (68.4%)	
(−)	15 (62.5%)	6 (31.6%)	
Vaginal odor *n* (%)			0.016
(+)	3 (19.6%)	7 (53.8%)	
(−)	19 (86.4%)	6 (46.2%)	
Vaginal irritation *n* (%)			0.013
(+)	8 (36.4%)	15 (75%)	
(−)	14 (63.6%)	5 (25%)	
Dyspareunia *n* (%)			0.15
(+)	15 (55.5%)	21 (72.4%)	
(−)	12 (44.5%)	8 (27.6%)	

**Table 4:** Comparison of vaginal cultures of participations with non-antimicrobial underwear.

Variables	Baseline Culture (*n* = 30)	Control Culture (*n* = 30)	*p* Value
Negative	16 (53.3%)	17 (56.6%)	
*C. albicans n* (%)	6 (20%)	5 (16.6%)	
*C. aurimicosum n* (%)	1 (3.3%)	0 (0%)	
*C. glabrata n* (%)	2 (6.6%)	3 (9.9%)	
*E. coli n* (%)	1 (3.3%)	1 (3.3%)	
ESBL *n* (%)	1 (3.3%)	1 (3.3%)	0.950
*G. vaginalis n* (%)	0 (0%)	1 (3.3%)	
*G. vaginalis* + *C. albicans n* (%)	1 (3.3%)	0 (0%)	
*P. mirabilis* + *E. coli n* (%)	1 (3.3%)	1 (3.3%)	
*S. agalactia n* (%)	1 (3.3%)	1 (3.3%)	

**Table 5:** Comparison of vaginal cultures of participations with antimicrobial underwear use.

Variables	Baseline Culture (*n* = 30)	Control Culture (*n* = 30)	*p* Value
Negative	12 (40%)	24 (80%)	
*C. albicans n* (%)	10 (33.3%)	1 (3.3%)	
*C. albicans* + *C. glabrata n* (%)	1 (3.3%)	0 (0%)	
*C. crusei n* (%)	2 (6.6%)	0 (0%)	
*C. glabrata n* (%)	1 (3.3%)	0 (0%)	
*C. glabrata* + *K. pneumonia n* (%)	1 (3.3%)	0 (0%)	
*E. coli n* (%)	1 (3.3%)	0 (0%)	
*E. coli* + *C. albicans n* (%)	0 (0%)	1 (3.3%)	0.018
*E. coli* + MRSA *n* (%)	1 (3.3%)	0 (0%)	
*E. coli* + *S. warneri n* (%)	0 (0%)	1 (3.3%)	
*K. aerogenes n* (%)	1 (3.3%)	0 (0%)	
*K. pneumonia n* (%)	1 (3.3%)	0 (0%)	
*S. agalactia n* (%)	0 (0%)	2 (6.6%)	

## Data Availability

The datasets used and analysed during the current study are available from the corresponding author on reasonable request.
